# South Dakota Veterinary Medical Association

**Published:** 1891-11

**Authors:** D. V. Capes

**Affiliations:** Secretary


*South Dakota Veterinary Medical Association.*—On Sept. 24th, 1891, several of South Dakota's veterinarians assembled in the parlor of the Cataract Hotel, Sioux Falls, South Dakota, and organized an association which will be known as the South Dakota Veterinary Medical Association.

The objects of this association are the mutual advancement of its members in veterinary science, the cultivation of fraternity, the elevation of the profession and the diffusion of the knowledge of veterinary medicine and surgery. After an hour's time passed in hand-shaking and making acquaintance, order was called. A constitution and by-laws were then read, adopted, and signed by those present, after which the following officers were elected: President, Prof. C. A. Cary, B. S., D. V. M., Brookings, S. D.; Vice-President, Dr. W. F. Heller, D. V. S., Sioux Falls, S. D.; Secretary, Dr. D. B. McCapes, V. S., Vermillion, S. D.; Treasurer, Dr. E. K. Paine, D. V. M., Sioux Falls, S. D.

The meeting then adjourned until next year when it is hoped there will be interesting topics before the association and much benefit derived.

D. V. Capes, V. S., *Secretary.*

